# From Concept to Commerce: Developing a Successful Fungal Endophyte Inoculant for Agricultural Crops

**DOI:** 10.3390/jof4010024

**Published:** 2018-02-11

**Authors:** Brian R. Murphy, Fiona M. Doohan, Trevor R. Hodkinson

**Affiliations:** 1 School of Natural Sciences & Trinity Centre for Biodiversity Research, Trinity College Dublin, The University of Dublin, College Green, Dublin 2, Ireland; hodkinst@tcd.ie; 2 UCD School of Biology & Environmental Science and UCD Earth Institute, University College Dublin, Dublin 4, Ireland; fiona.doohan@ucd.ie

**Keywords:** barley, commercialisation, crop wild relatives, endophytic fungi, yield enhancement

## Abstract

The development of endophyte inoculants for agricultural crops has been bedevilled by the twin problems of a lack of reliability and consistency, with a consequent lack of belief among end users in the efficacy of such treatments. We have developed a successful research pipeline for the production of a reliable, consistent and environmentally targeted fungal endophyte seed-delivered inoculant for barley cultivars. Our approach was developed de novo from an initial concept to source candidate endophyte inoculants from a wild relative of barley, *Hordeum murinum* (wall barley). A careful screening and selection procedure and extensive controlled environment testing of fungal endophyte strains, followed by multi-year field trials has resulted in the validation of an endophyte consortium suitable for barley crops grown on relatively dry sites. Our approach can be adapted for any crop or environment, provided that the set of first principles we have developed is followed. Here, we report how we developed the successful pipeline for the production of an economically viable fungal endophyte inoculant for barley cultivars.

## 1. Introduction

The use of microorganisms to improve agricultural crop performance has a long history, but only in the last few decades have we been able to describe in any detail how bacteria, fungi, algae and protozoans interact with plants to enhance desired traits [1,2,3]. Mankind has understood the benefits of various practices, often without conscious intent, that directly or indirectly involve the manipulation of the soil and plant microbiota [4,5]. Only in the last century have we incorporated the use of microorganisms in agriculture in a deliberate, focused and knowledgeable way [6,7]. However, it is the plant that is the ultimate arbiter of how and when these microorganisms are incorporated into the functional plant microbiome, often in an unpredictable fashion [8,9]. It is the goal of all plant-microbiome research to understand and use these relationships in the most effective way, but we are still some way from obtaining this full understanding [10,11]. Such a lack of informed and validated knowledge has resulted in inconsistent results when using microorganisms in the field situation, resulting in a reduced end-user confidence and uptake of the technology [12,13].

Endophytes are a class of plant-associated microorganisms that have shown particular promise in agriculture [14,15,16,17,18,19,20,21]. Endophytes (bacteria, fungi and unicellular eukaryotes) live at least part of their life cycle inter- or intracellularly inside plants, usually without inducing pathogenic symptoms. This can include competent, facultative, obligate, opportunistic and passenger endophytes (a passenger endophyte enters the plant by accident in the absence of selective forces maintaining it in the internal tissue of the plant [22]). Endophytes can have several effects on plants and/or may change function during their life-cycle [14]*.* Bacterial and fungal endophytes have shown promise as beneficial crop inoculants, and many are known to enhance abiotic and biotic plant stress tolerance [23,24,25,26,27,28]. But a large proportion of the associated studies have been conducted in a controlled environment and do not translate successfully to the field [29].

The approach adopted by our research group has consciously focused on making this transition from ‘pot to plot’ more successful. We aimed to remove the inconsistency from endophyte application in field crops by targeting a single crop, barley, as our model plant, and testing the efficacy of endophyte strains under various environmental stresses, culminating in extensive field trials [30,31,32,33,34,35]. Only with this field validation can we say with any confidence that a microbial inoculant really “works” [36]. We have demonstrated the effectiveness of this approach by developing a fungal endophyte inoculum that consistently increases barley grain yield over several seasons under a variety of chemical fertilizer inputs in dry growing environments [31]. The outcomes from our research and field application have been so successful that we have been able to bring our endophyte technology into commerce, which will result in significant reductions in economic and environmental costs.

This article will review the development of this technology, outlining the concepts involved and the methods employed to create a viable, consistent and environmentally targeted commercial endophyte inoculant for barley crops. We will discuss a set of first principles that are important for success, and we will outline future research directions that will increase the portfolio of endophyte inoculants and expand the scope of target crop species.

## 2. Concept

Before any resources are allocated to an endophyte research project, it is important to understand the problem that is being addressed and the solution that is proposed. The problem must be a real commercial and/or functional need of the eventual user of the solution. Nearly half of all company start-ups fail because there was no market need for the product [37]. So it is essential that the proposed concept is going to provide a viable and needed solution. This solution should also attract potential commercial partners and collaborators on the project, thus increasing the resources available for successful implementation. For illustration, we focused on the need to reduce the high economic cost of chemical crop inputs; any product that successfully addresses this need would provide real and tangible benefits for the crop growers and also reduce environmental impact of the chemical inputs.

Crop wild relatives are an important genetic resource for breeding desired plant traits into related crop cultivars [38]. We suspected that the same was true for microbial resources, and particularly for fungal endophytes. Our hypothesis was that endophytes that were recoverable from crop wild relatives would be more compatible with the related crop, both for inoculation and colonization purposes and for *in planta* behaviour. We further hypothesized that endophytes recovered from a particular type of environment would be more compatible and effective on crops growing in a similar environment, and that endophyte crop wild relative interactions were more likely to have been maintained through time than endophyte-free elite crop cultivars.

It was important to retain this focus in all subsequent experiments so that we could produce a set of results that may or may not support our hypotheses. We were also aware that to produce a comprehensive and useful dataset, with the resources available, we would need to target only one important crop species. For our purposes, barley was the ideal crop as it is the most important arable crop grown in Ireland, where it is grown on over 200,000 ha, and there are several local wild barley relatives available for sampling.

The endophyte source we selected was the wild barley species *Hordeum murinum* ssp. *murinum* L. (wall barley), as it is relatively common and occurs in a wide variety of habitats on the east coast of Ireland, especially those subject to human disturbance. This enabled us to sample from diverse environments and to establish any significant correlations between endophyte recovery and alpha/beta diversity. It also gave us the largest possible set of endophyte strains from which to screen potential study taxa. The only other naturally occurring wild barley species in Ireland are *Hordeum jubatum* and *Hordeum secalinum,* and these only occur in rare and scattered populations [39].

By targeting endophytes recovered from natural and native plant populations we avoid introducing exotic microorganisms into the environment which may face regulatory and safety issues. They would also likely be more suited to local barley crop growing conditions, as they would have been selected by historical climatic conditions. Finally, as these host plants and geographical locations have never previously been sampled for fungal endophytes, we expected that the strains would be novel and previously uncharacterized, a real advantage when there is a need to protect intellectual property.

## 3. Developing the Concept

### 3.1. Endophyte Recovery

Having selected the barley wild relative to sample, it was important to ensure that we could recover the greatest variety of endophytes for subsequent biofertilisation and biocontrol screening in agricultural applications. Here, we followed a set of general principles derived from years of experience, and which we recommend for similar research.

The most important of these was the selection of the sampling sites, as this will determine the direction and success of all subsequent work:

- Sampling site should contain a minimum population of 10 genetically individual plants
- Plants on sampling site should be healthy and free of disease
- If selected for biotic tolerance, the site should preferably be in an area of potentially high disease pressure for the species
- There should be some form of abiotic stress present at the site, such as a very dry or nutrient-poor soil, which must be measureable
- The site should be relatively undisturbed and natural, i.e., not recently worked
- The site should not be exposed to contaminants, such as close to a busy roadside
- The site should preferably contain no alien plant species
- The host plant species should be identifiable using a recognised and reliable key
- The plants should be actively growing and not in a senescent phase
- The plants should be sampled when the site is in its ‘normal’ condition, i.e., not after any heavy rain, frost or unusual weather event, nor after any disturbance

While collecting the plant material it is vital to obtain as much environmental data as possible, as this information will be the major deciding factor when selecting individual strains for consortia application at the crop growing sites. For example, endophyte strains that are sourced from a particularly dry site may be more suitable for a relatively drought-stressed crop growing environment. Recording of environmental data should include variables such as GPS location, soil pH, soil moisture, soil nutrients, soil salinity, soil type, accompanying ground vegetation, tree cover, and exposure. We also recommend collecting a soil sample onto dry ice for later analysis of soil microbial community and elemental constituents. It is also important to ensure that plant sampling is aligned with principles outlined in The Nagoya Protocol on Access to Genetic Resources and the Fair and Equitable Sharing of Benefits Arising from their Utilization to the Convention on Biological Diversity, also known as the Nagoya Protocol on Access and Benefit Sharing (ABS) [40].

For our experimental work, we chose endophyte source sites that were particularly dry, with thin sandy soils and relatively high soil pH and salinity (Table 1). These sites were chosen as we ultimately wanted to test the effects of the endophytes on field barley crops growing in similar environments; so we had the experimental goal in mind at all times from initial concept to pre-planning to field sampling. This goal-oriented approach is at the heart of our methodology, and can be applied to the selection of endophytes for other environmental conditions.

The plant tissue that is chosen for sampling must be disease- and blemish-free, especially when sampling roots. The sampled tissue should be placed in sealed plastic bags immediately and processed in the laboratory as soon as possible, preferably within one day of sampling. Well-established protocols can then be followed for inoculation and incubation of the plant tissue to enable maximum recovery of endophyte isolates. A general principle to follow when preparing the culture media is to use the medium concentrate at only 50% of the manufacturers’ recommendation, i.e., double the proportion of water. This will reduce the chances that the endophyte will experience any osmotic shock when emerging from the plant tissue and also make the culture medium more open and accessible for explorative hyphae. We also found that adding a high proportion (50%) of autoclaved plant extract increased the number of endophyte isolates recovered [41], an effect also reported by Prior et al., 2014 [42]. This plant extract should ideally be obtained from the same source species that is being sampled for endophytes. The endophytes are thus emerging into a chemically similar environment to that of the plant interior. Emerging hyphae need to be subcultured immediately, and sometimes single spore or hyphal culturing is required.

Identification of the isolates can be carried out using a combination of standard morphological and genetic barcoding techniques with analytical tools [43]. Establishing the endophyte strain identity is the most important process in screening the isolates for future experimental work.

### 3.2. Endophyte Screening

The initial recovery and isolation of endophytes from the host populations is only the first step in a comprehensive screening process to select the isolates with the greatest potential as crop inoculants. We took several factors into account when selecting strains for efficacy testing with barley crops:

1. The isolate should not be related to any known human or plant pathogen. This is a crucial factor which needs careful qualifying. The degree of DNA similarity between isolate and closest match is important in deciding the identity and whether to proceed with the isolate. Genetic similarity can best be judged by comparing a standard barcoding gene from the isolate with known accessions deposited in a genetic database such as GenBank (NCBI). For our purposes we compared the nuclear ribosomal Internal Transcribed Spacer gene (ITS), as it is sufficiently discriminatory to the species level when assigning taxonomy [44,45,46,47,48,49]. If the isolate is only distantly related to a known pathogenic strain, then it may be worth pursuing but is a personal judgement call depending on taxonomic group. Fungal isolate growth and proliferation at 37 °C would also be a contra-indicator for selection.
2. The isolate must show vigorous growth on a range of substrates.
3. The isolate should produce early and copious spores (endophyte cultures that do not easily produce spores are of limited use in large-scale agricultural applications).
4. The isolate should be pure; i.e., subcultured from a single spore.

It is likely that the sampling of host populations will result in a large collection of endophyte isolates and final selection of which isolates to use in subsequent experiments will depend largely on the resources available—it may be unrealistic to test 100 s or 1000 s of individual isolates. Careful screening and selection at this point will enable a more focused approach, and the controlled environment experiments will enable a further reduction of isolates selected for field trials. So we recommend that a set of criteria based on desired strain characteristics be used to select the final experimental subjects. For example, fungal endophytes should grow readily on a broad range of substrates, be of easy culture, retain competence over many cycles of sub-culturing, and should produce copious spores/conidia at a relatively early stage after initial recovery and culture. We have found that relatively few strains pass these criteria; from over 100 recovered strains, only 12 were finally selected for further experimentation [50].

## 4. Proof of Concept

With the basic ideas now in place we proceeded to obtain a proof of concept for our approach. At this stage, we have a selection of identified endophyte strains collected from a particular environment. Before using these strains in experimental work, the target agronomic traits must be clear from the very start, and the statistical analyses that will be used should be established; as the eminent statistician Sir Ronald Fisher once said: ‘To consult the statistician after an experiment is finished is often merely to ask him to conduct a post mortem examination. He can perhaps say what the experiment died of [51]. There is always a danger that any post-hoc selection of statistical methods may bias the analyses in favour of the desired results [52].

For many agricultural crops, yield is often the most important trait of interest and is related to a whole plethora of influences: pathogen and disease resistance, nutrient use efficiency, photosynthetic efficiency, hormone and metabolite balance, abiotic stress resistance, etc. So it makes sense to test the effect of the endophyte inoculant on as many of these factors as possible; there is no short cut to developing an effective endophyte inoculant. The crucial point here is to focus on the main influences on crop performance that will be experienced by the target crop species in a particular environment.

To achieve a comprehensive set of data for barley-endophyte responses under different conditions, we decided to carry out extensive controlled environment experiments under a variety of stresses using the fungal endophytes recovered from the sampled populations of *H. murinum*.

Prior to the experimental cycle, a range of commercial culture media combined with whole plant extract of *H. murinum* were tested for their effect on endophyte recovery from the roots of their host (*H. murinum*), and for their subsequent growth and sporulation [41]. It was found that there were significant differences between the media in endophyte recovery, endophyte mycelial growth and time to sporulation. A significantly greater number of different endophytes were recovered from roots on the malt extract plus whole plant extract medium than any of the other media tested. These results indicated that different media are suitable either for the initial recovery and isolation of fungal root endophytes or for increasing fungal biomass and inducing earlier sporulation.

The first controlled-environment experiment with barley examined the effects of ten endophyte strains on disease development on seeds of a barley cultivar using five artificial and one soil-based growth media [30]. A co-inoculant of all ten isolates, as well as two individual isolates, successfully suppressed the development of seed-borne fungal infections on germinated and ungerminated seed. We further reported that the ability to suppress the seed-borne infections was related to the soil properties of the isolate origin [33].

Secondly, we found that inoculation with six different individual endophytes increased grain yield in a nutrient-starved barley cultivar by up to 29% [32]. Furthermore, we also showed that inoculation with the isolates induced increases of up to 70% in shoot dry weight in the nutrient-starved spring barley; the nutrient input was only 10–15% of that recommended. 

Thirdly, five individual endophyte strains induced significant improvements in agronomic traits for a severely drought-stressed barley cultivar, including the number of tillers, grain yield and shoot biomass [35]. Soil moisture content was allowed to reach 10–15% of field capacity before watering. The trait that showed the greatest significant difference in the drought-stressed plants was the number of tillers, where all of the endophyte treatments induced a greater number of tillers per plant. However, except in one case, the mean dry root weight for all plants was greater in the control plants, indicating preferential allocation of resources to above-ground parts in the endophyte treatments.

Fourthly, five fungal root endophytes, either individually or combined, were inoculated onto seeds of a barley cultivar grown in optimal conditions and under a combined drought (10–15% soil moisture), heat (33 °C), nutrient (15% of recommended input) and pathogen (*Gaeumannomyces graminis*) stress (multiply-stressed). We found a greater endophyte-induced improvement in important agronomic traits in the multiply-stressed plants compared with the plants grown in optimal conditions [53]. For the multiply-stressed plants, only 13% of the controls survived to the end of the experiment compared with 80% of the endophyte treatments. In multiply-stressed plants, the endophytes induced increases in the number of tillers and root and shoot biomass. The improvements were most significant for barley inoculated with a combination of all five endophytes.

Finally, we tested a range of seed dressings to evaluate the effects of the endophytes on germination and early growth of barley. Seeds were either untreated, dressed with a fungicide (triticonazole and prochloraz), an endophyte spore solution or a combination of endophyte and fungicide. We found significant increases in mean barley seedling length induced by the endophytes after 28 days of seedling growth at time points of up to 6 months from seed dressing. The increases in mean seedling length were greatest for the combined fungal endophyte and fungicide treatment. These results indicated that the endophytes tested were persistent in a seed dressing, enhanced early seedling growth and were fungicide tolerant [34].

Results from these experiments showed that novel symbiotic associations between barley and fungal root endophytes significantly increased yield and biomass in barley grown under nutrient, drought, heat and pathogen stress, and also suppressed the development of seed-borne pathogenic infections.

## 5. Translation

Isolating, selecting and testing the endophytes in controlled environments to determine their potential as crop inoculants is only the first step towards future agricultural application. We now needed to see whether the positive results translated to field grown barley. The four endophyte strains that induced the greatest significant yield increase in nutrient- and drought-stressed barley were selected as the consortium members. We reasoned that the different modes of action associated with each of the two stresses would allow a degree of compatibility as a consortium, with each endophyte bringing different functional mechanisms to the plant in a particular ecological niche space [54,55,56,57,58].

To obtain the maximum benefit from the endophytes as a crop treatment, it is essential to select the most effective inoculant delivery formulation. The formulation will depend to a large extent on the type of fungal material that is to be used as the active ingredient. The method of applying the treatment should be economically and ergonomically viable, and be easy to apply. The inoculant carrier substrate should provide a stable environment for the microbial fractions and prolong product shelf-life [59]. Some microbial products have a very short shelf-life [12], with a severely reduced window of effectiveness. Our decision to dress the barley seeds with environmentally stable fungal endophyte spores helps to provide robustness and preserve vitality of the treatment, even several months after dressing [34]. Dressing the seeds using standard seed dressing equipment means that the seed supplier does not need to purchase extra and possibly expensive equipment.

Dressing the seed also ensures that the endophyte inoculant will be deployed in the most efficient way, as the inoculant is delivered precisely to where it will be most effective, with no wastage. The germinated seedling root(s) will come into immediate contact with the fungal spores, which will then colonise the plant at the earliest possible stage. This will also have the added benefit of reducing the release of the strains into the environment. The endophyte may be more effective if it colonises the plant tissue before any competitors, as the order of arrival of endophyte and pathogen may even change the effect on the plant from beneficial to detrimental [60].

This scenario depends on a productive relationship with industry partners, and this is often the point at which transition from promising laboratory results to real-world applications fails. Unless specific funding is available to support this process then the researcher must rely on the good will of the industry partner(s). Getting the right partners in the right place at the right time will have a big effect on the success of the translational research effort. We have found it helpful to include such funding in the associated research proposal. This is particularly important when trialing field crops, as multi-year validation is required before any robust claims can be made for the effectiveness or otherwise of the endophyte treatment.

The approach we have adopted in developing a crop inoculant, as outlined in this paper, has resulted in the successful translation of promising laboratory-based research to agriculture. In field trials with barley, we found that for an endophyte-associated increase in grain yield, a strong correlation was found between increased yield and low seasonal rainfall [31]. Furthermore, the endophytes were just as effective with regular foliar fungicidal treatment. Another recently completed field trial on dry sites also returned significant endophyte-induced yield increases (manuscript in preparation), providing strong validation for the value of our methodology.

## 6. Discussion

**Figure 1:**
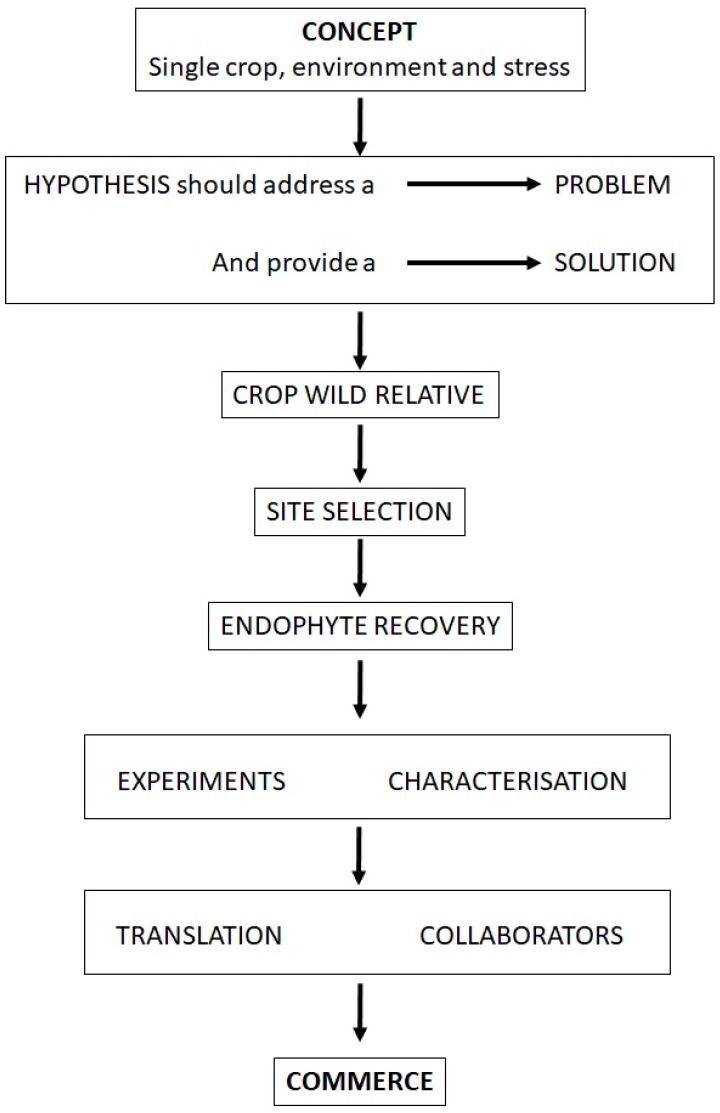
From Concept to Commerce: a schematic flowchart for the prospecting of endophytes for use in agriculture.

Many promising scientific discoveries do not make a successful transition to commerce, for any number of reasons, while some discoveries happen almost by accident [61]. The most common point of failure may be in the initial research project design, where a focused, achievable and outcome-based plan is not in place [62]. This is especially true of research into the potential of endophytes in agricultural crops, where many valuable discoveries languish in the limbo between the laboratory bench and the farmer’s field. In this paper, we have shown that a properly focused endophyte project with a continuous emphasis on one particular outcome can result in a more successful transition from idea to application. We have described a set of best practices leading to the development of a successful endophyte field crop inoculant, and this approach can, in principle, be adapted for any crop or agricultural environment (including marginal lands) provided that a similar methodology is followed. The flowchart (Figure 1) shows the basic steps and decision points of our approach.

The prospecting and discovery of endophytes that have beneficial application to agricultural crops is an ongoing and potentially open-ended enterprise, with rates of successful application only limited by available resources and unproven methodologies. Resources are finite and competitive, but ensuring that the methods and principles applied are sound will maximize the chance of success. While the use of endophytes as beneficial crop inoculants has potential, the solution to a defined agricultural need is often complex and multi-factorial and it is unlikely that a single approach will provide the full answer. Future research directions should focus on combining different approaches that will provide more robust and persistent solutions for agriculture. For instance, an endophyte crop treatment combined with a change in agricultural practice can give extra benefits. For example, moving towards a ‘no-till’ method of cultivation along with longer rotations and the use of cover crops can drastically reduce chemical inputs and labour costs [63] and, if combined with an endophyte inoculant, may produce surprising synergistic results.

Global climate change will bring new and greater stresses to bear on crops, and future research efforts should focus on the solutions to the expected changes. It may be best to target expected conditions in particular geographic areas experiencing particular stresses, especially in poorer areas where farmers cannot afford to use expensive chemicals. Endophyte-based solutions to stress-related problems that minimize economic and environmental costs have the potential to bring real benefit to those who need it the most.

## Figures and Tables

**Table 1:** Soil variables for endophyte recovery sites.

Location	pH	Moisture Content %	Salinity *	Soil Type
1	7.8 ± 0.0	9.1 ± 0.0	1.18 ± 0.0	Loamy silt, stony
2	8.0 ± 0.0	13.4 ± 0.0	1.41 ± 0.0	Loamy silt, shallow
3	7.6 ± 0.0	0 ± 0.0	1.22 ± 0.0	Sandy silt, shallow
4	7.9 ± 0.0	4.4 ± 0.0	1.39 ± 0.0	Sandy silt, stony
5	7.9 ± 0.0	0 ± 0.0	1.45 ± 0.0	Sandy silt, shallow
6	7.7 ± 0.0	19.5 ± 0.0	1.26 ± 0.0	Clay loam, shallow
7	7.7 ± 0.0	0 ± 0.0	1.49 ± 0.0	Sandy silt, shallow
8	7.7 ± 0.0	0 ± 0.0	1.51 ± 0.0	Sandy silt, shallow
MEANS	7.8	8.3	1.37	

* Salinity is Osmotic Pressure in bars. pH, Moisture content % and Salinity are mean values ± standard error (*n* = 10).
